# Electroconvulsive Therapy’s use in Idiopathic Intracranial Hypertension with Mood Disorder: caution, promise, and progress

**DOI:** 10.1192/j.eurpsy.2022.769

**Published:** 2022-09-01

**Authors:** M. Parker, B. Carr

**Affiliations:** University of Florida College of Medicine, Department Of Psychiatry, Gainesville, United States of America

**Keywords:** mood disorder, Idiopathic Intracranial Hypertension, Pseudotumor Cerebri, ECT

## Abstract

**Introduction:**

Idiopathic Intracranial Hypertension (IIH) is a condition characterized by an increase of intracranial pressure (ICP) with no identifiable cause to date. One-half of patients who suffer from IIH have co-morbid mood disorders, such as Major Depressive Disorder (MDD), that can be refractory to pharmacologic treatment. Electroconvulsive Therapy (ECT) is a safe and effective treatment for treatment-refractory mood disorder, but possesses a relative contra-indication for IIH due to its theoretical increase in ICP. Can ECT become the gold-standard treatment modality for mood disorder from IIH?

**Objectives:**

We aim to synthesize and summarize the state of the literature surrounding the intersection of ECT and IIH. We will present notable findings and propose avenues for future investigation.

**Methods:**

We conducted a literature review using PubMed’s search function. Key terms that were queried are as follows: Idiopathic Intracranial Hypertension, Pseudotumor Cerebri, Benign Intracranial Hypertension, Mood Disorder, Major Depressive Disorder, ECT, Electroconvulsive Therapy.

**Results:**

The prevailing theory of IIH and mood disorder centers around HPA axis dysfunction, which has been heavily theorized to be positively impacted with ECT. ECT itself may not increase the ICP, but the anesthesia might. The only two case reports in the literature presented safe and successful use of ECT’s in patients with IIH and MDD.

**Conclusions:**

More data is needed to draw conclusions, as the literature surrounding ECT’s use in patients with IIH remains sparse. Further studies must explore whether ECT’s use in IIH remains effective. Through this, we may understand more about both IIH and ECT itself.

**Disclosure:**

No significant relationships.

